# Comprehensive Analysis of NPSR1-AS1 as a Novel Diagnostic and Prognostic Biomarker Involved in Immune Infiltrates in Lung Adenocarcinoma

**DOI:** 10.1155/2022/2099327

**Published:** 2022-10-15

**Authors:** Hui Zhang, Jin Yuan, Yuehua Xiang, Yong Liu

**Affiliations:** ^1^Department of Pulmonary and Critical Care Medicine, The Central Hospital of Enshi Tujia and Miao Autonomous Prefecture, Enshi, China; ^2^Department of Pulmonary Disease Diabetes Mellitus, The National Hospital of Enshi Autonomous Prefecture, Enshi, China

## Abstract

The incidence of lung adenocarcinoma (LUAD), the most common subtype of lung cancer, continues to make lung cancer the largest cause of cancer-related deaths worldwide. Long noncoding RNAs (lncRNAs) have been shown to have a significant role in both the onset and progression of lung cancer. In this study, we aimed to investigate the clinical significance and underlying mechanism of lncRNA NPSR1-AS1 (NPSR1-AS1) in LUAD. First, we performed an analysis on TCGA and identified 229 differentially expressed lncRNAs (DELs) (including 216 upregulated lncRNAs and 13 downregulated lncRNAs). Then, we carried out a screening of the lncRNAs associated with survival, and a total of 382 survival-related lncRNAs were found. 15 survival-related DELs were identified. Among them, our attention focused on NPSR1-AS1. We found that the expression of NPSR1-AS1 was much higher in LUAD specimens compared to nontumor tissues. According to the results of the ROC assays, high NPSR1-AS1 expression had an AUC value of 0.904 for LUAD, with a 95% confidence interval ranging from 0.881 to 0.927. The expression of NPSR1-AS1 was shown to be significantly elevated in a wide variety of cancers, according to the findings of a pancancer investigation. Functional enrichment analysis confirmed that NPSR1-AS1 was involved in LUAD progression via regulating several tumor-related pathways. Patients with high levels of NPSR1-AS1 expression were shown to have a shorter disease-specific survival (DSS) or overall survival (OS) than those with low levels of NPSR1-AS1 expression, according to the findings of a clinical investigation. It was determined by multivariate analysis that NPSR1-AS1 expressions served as an independent prognostic factor for the overall survival of LUAD patients. The results of immune cell infiltration revealed that the expressions of NPSR1-AS1 were negatively associated with CD8 T cells, pDC, cytotoxic cells, mast cells, iDC, neutrophils, NK CD56dim cells, DC, Th17 cells, Tgd, and macrophages, while they were positively associated with NK CD56bright cells and B cells. Overall, our findings revealed that NPSR1-AS1 could serve as a potential biomarker to assess the clinical outcome and immune infiltration level in LUAD.

## 1. Introduction

It is estimated that 1.76 million people die every year from lung cancer, making it the top cause of death resulting from cancer worldwide (18.4% of all cancer-related deaths) [1]. Approximately forty percent of all instances of lung cancer are classified as lung adenocarcinoma (LUAD), making it the most frequent type [2, 3]. The frequency of this form of lung cancer is rising worldwide. The primary cause of lung cancer is still smoking, as it has been for decades [4, 5]. Even though prolonged exposure to tobacco smoke is by far the most common cause of this form of cancer, nonsmokers account for anywhere from 15 to 20 percent of cases and are typically thought to have contracted the disease due to a confluence of hereditary and environmental factors [6, 7]. Despite the use of morphological analysis to classify patients into different risk groups, it is evident that the overall survival (OS) rates for LUAD patients with high invasiveness and early metastasis ranged from 13 to 58.3% at 5 years [8, 9]. This was the case despite the use of morphological analysis to classify patients. It is difficult to diagnose non-small-cell lung cancer in its early stages, when the disease is also tough to treat [10]. Consequently, it is of the utmost importance and a pressing necessity to discover innovative prognostic biomarkers in order to provide helpful therapy methods for LUAD.

The discovery of the potential diagnostic usefulness of genetic biomarkers, such as long noncoding RNAs (lncRNAs), was made possible by the advent of high-throughput sequencing techniques and bioinformatics technologies [11, 12]. lncRNAs are becoming an increasingly important focus of attention in research on cancers [13]. lncRNAs are a category of nonprotein coding transcripts that are typically longer than 200 nucleotides without an open reading frame [14]. Their length is what defines them as “long noncoding RNAs.” Numerous studies have shed light on the function of lncRNAs in several biological processes, such as the silencing of X-chromosome genes, the remodeling of chromatin, and transcriptional activity [15, 16]. In addition, a growing number of studies have indicated a link between lncRNAs and the onset and progression of many malignancies, including LAUD [17, 18]. For instance, Zhang et al. indicated that the expressions of SNHG17 were highly elevated in LUAD specimens and cells, and high SNHG17 expression was related to advanced stages of tumor node metastases and a bad prognosis for patients who had LUAD. The targeting of the microRNA-193a-5p/NETO2 axis by SNHG17 knockdown resulted in an inhibition of the EMT process as well as cell migration, invasion, and proliferation [19]. Cong et al. showed that it is possible that the lncRNA known as linc00665, which was found to be significantly overexpressed in lung adenocarcinoma (LUAD) tissues, can act as an independent predictor of a bad prognosis. According to the results of functional tests, linc00665 promoted LUAD cell proliferation and metastasis both in vitro and in vivo through modulating the AKR1B10-ERK signaling pathway and by sponging miR-98 [20]. These studies suggested that lncRNAs have the potential to be turned into potentially valuable biomarkers that can aid in the diagnosis and prognosis of LUAD.

Although immunotherapy has been used with promising results in the treatment of tumors, it is still only effective for a very small percentage of cancer patients [21]. This is despite the fact that it represents a unique approach to cancer treatment [22]. There is a strong correlation between the tumor microenvironment (TME) and the effectiveness of immunotherapy [23, 24]. The epigenetic differentiation of tumor cells and the metastasis and infiltration of the tumor are both linked to the suppression of the immune system caused by the tumor [25]. TME is a complex system that is made up of many distinct cell types, cytokines, and other extracellular components. Both the kind and the quantity of immune cells that invade a tumor are significant factors in establishing its development and evolution [26, 27]. Additionally, the make-up and proportion of TIICs and stroma can be used for the diagnosis, prognosis, and prediction of many cancers [28]. It is possible that new therapy targets for cancers could be found by mining related lncRNAs and then examining how those lncRNAs affect immune cell infiltration in TME and the prognosis of the tumor.

In this study, NPSR1-AS1, a previously unknown long noncoding RNA associated to LUAD, was found to have abundant expression in LUAD. Previous researches from a number of different investigations have uncovered its roles in some cancers. For instance, Ni et al. revealed that NPSR1-AS1 was substantially expressed in thyroid cancer, and its overexpression boosted the proliferation and metastasis of thyroid cancer cells. It was accomplished by recruiting ELAVL1 to stabilize NPSR1 mRNA [29]. NPSR1-AS1 was found to be highly expressed in thyroid cancer. On the other hand, its expression and potential prognostic usefulness in LUAD have not been researched. The investigation of the immunological microenvironment in patients with LUAD has opened up new possibilities for the conventional therapy protocols that are now in use. Therefore, the improvement of patient survival is one of our primary objectives in the development of a universal immunodiagnostic marker.

## 2. Materials and Methods

### 2.1. Patient Datasets

Using the UCSC Xena browser, we were able to retrieve the gene expression data, phenotypic data, and extensive clinicopathological data for TCGA-LUAD. The Illumina HiSeq RNA-Seq platform was used to retrieve the sequence data that was needed. For the purposes of the subsequent studies, the HTSeq-FPKM gene expression data were converted into TPM. TPM produces results that are more comparable to those provided by an approach using microarrays, and it makes it easier to compare the results of different samples. In accordance with the associated annotation file, the probe ID was transformed into the gene symbol, and then, the average expression values for many probes that corresponded to the same gene were computed. The data were collected and analyzed in a way that was compliant with the publication standards provided by TCGA datasets. There was not a single study that directly involved human volunteers or animal testing that was included. The approval of the ethics committee and informed consent were not required.

### 2.2. Identification of Differentially Expressed lncRNAs (DELs)

All samples were compared using a differential expression analysis between LUAD and nontumor samples, and the Wald significance test (as specified by the nbinom Wald test function) was utilized to determine statistical significance. RNA-seq data can be trusted when analyzed using the DESeq2 package in R, which employs this test. This package was based on raw read counts for each gene, making it a robust way for assessing RNA-seq data. A statistical limit for significance was set at a false discovery rate (FDR) of less than 0.05 and a fold change of more than 4.

### 2.3. Survival Analysis

A high-expression group was defined as having an expression level that was higher than the median expression level across all samples, and a low-expression cohort was defined as having an expression level that was lower than the median expression level across all samples. It was performed in order to facilitate the screening process for survival-related genes. A log-rank test was applied to compare the Kaplan-Meier curves of the high-expression cohort with those of the low-expression cohort. In order to determine the factors that are linked with survival, a multivariate analysis using the Cox proportional hazard model was carried out. Adjusted hazard ratios (HRs) and 95% confidence intervals (CIs) are reported. The level of significance for each test was two-sided and set at *P* less than 0.05. The “survival” package in R was utilized in order to successfully complete the procedure (https://cran.r-project.org/web/packages/survival/index.html).

### 2.4. Functional Enrichment Analysis

We separated the tumor groups into high- and low-expression subgroups based on the median expression values of NPSR1-AS1, and we used the “limma” R package to screen for differentially expressed genes (DEGs) between the two subgroups. In order to be considered statistically significant, the |logFoldChange (logFC)| value needed to be greater than 2, and the false discovery rate (FDR) needed to be lower than 0.05. The next step was to conduct a study of GO and KEGG enrichment of MMP14 coexpressed genes. The procedure was carried out with the assistance of the R programming language and the clusterProfiler, Enrichment plot, and GGplot2 software programs.

### 2.5. Analysis of Infiltrating Immune Cell Types (TIICs) in the Microenvironment of LUAD

To examine the relative expression levels of 22 different TIICs in LUAD samples, the CIBERSORT package of the R software was utilized in its version 3.6.3 form [30]. We determined the percentages of each of these 22 TIIC subpopulations that were present in each sample.

### 2.6. Statistical Analysis

The statistical studies were carried out with the help of the R programming language. The Wilcoxon test was used to evaluate whether or not there were continuous variable differences between the two groups. The Kruskal-Wallis test was applied to make comparisons between more than two different groups. Either the chi-square test or Fisher's exact test was used to investigate the variations in frequency of occurrence between category variables. The log-rank test was utilized for the study of the variations in survival rates. In the analysis of disease-specific survival (DSS) or overall survival, the Cox assays was utilized for the purpose of calculating the hazard ratios (HRs) of variables together with their respective 95% confidence intervals (95% CIs). Moreover, Pearson's correlation and Spearman's correlation were used to assess the correlations between different genes. A *P* < 0.05 was considered statistically significant.

## 3. Results

### 3.1. Identification of the Survival-Related DELs in LUAD

**Figure 1:**
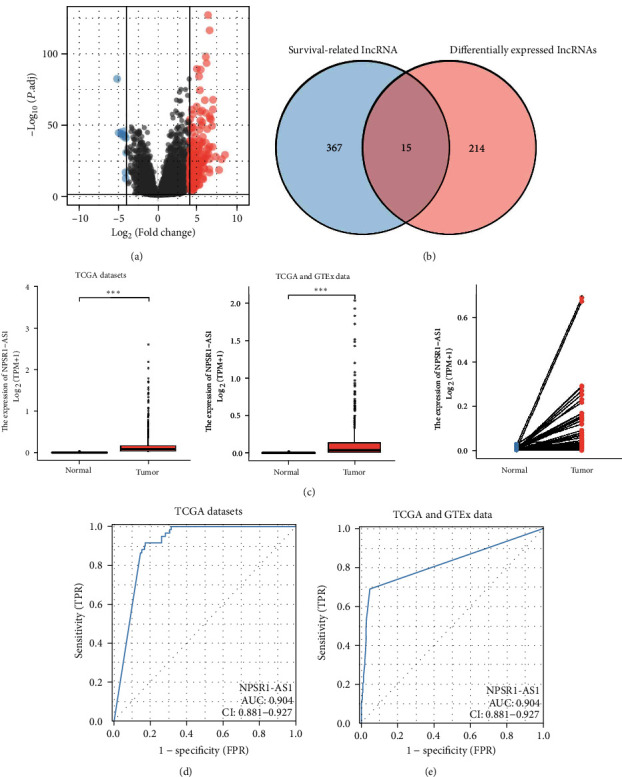
The expression of NPSR1-AS1 was distinctly increased in LUAD. (a) 229 DELs (including 216 upregulated lncRNAs and 13 downregulated lncRNAs) between LUAD specimens and nontumor specimens from TCGA datasets were shown in volcano map. (b) Venn diagram showed the overlapping lncRNA between 229 DELs and 382 survival-related lncRNAs. (c) The expression of NPSR1-AS1 in LUAD specimens and nontumor specimens. (d, e) ROC assays were used to investigate the diagnostic value of NPSR1-AS1 for LUAD patients in the datasets obtained from TCGA.

The transcriptional profiles of 535 tumor samples and 59 normal samples were first retrieved from TCGA databases and then reanalyzed by our team. It was possible to determine the levels of expression for all lncRNAs. We were able to collect a total of 229 DELs, 216 of which were upregulated lncRNAs and 13 of which were downregulated lncRNAs (Figure 1(a)). Then, we screened the lncRNAs associated with survival, and we found 382 survival-related lncRNAs that had a *P* value of less than 0.01 (Table S1). Venn diagram showed the overlapping lncRNAs between 229 DELs and 382 survival-related lncRNAs, and 15 survival-related DELs were identified, including FAM83A-AS1, LINC01833, LASTR, AC022784.1, AC068228.1, AC010343.3, NPSR1-AS1, LINC01559, AC005256.1, AC125603.2, AL365181.3, AL365181.2, AC125603.1, LINC00973, and LINC02535 (Figure 1(b)). NPSR1-AS1 was the primary focus of our study among the 15 survival-related DELs listed above. We observed that the expressions of NPSR1-AS1 were markedly elevated in LUAD tissues when compared to nontumor specimens (Figure 1(c)). Additionally, the diagnostic significance of NPSR1-AS1 for LUAD patients was investigated using data from TCGA datasets. According to the results of the ROC tests, high NPSR1-AS1 expression yielded an AUC value of 0.904 for LUAD with a 95% confidence interval ranging from 0.881 to 0.927 (Figure 1(d)). Moreover, based on the data from TCGA and GTEx data, the results of ROC assays indicated that high NPSR1-AS1 expression had an AUC value of 0.824 (95% CI: 0.801 to 0.847) for LUAD (Figure 1(e)).

### 3.2. Pancancer Analysis of NPSR1-AS1 Expression

**Figure 2:**
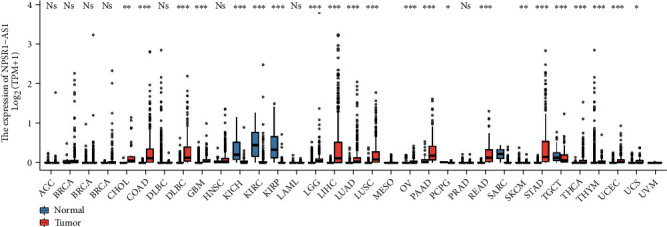
Pancancer analysis of NPSR1-AS1 expression.

In addition, we performed pancancer analysis of NPSR1-AS1 expression using TCGA and GTEx data. As shown in Figure 2, we found that the expression of NPSR1-AS1 was distinctly increased in many types of tumors, such as CHOL, COAD, and ESCA. Our findings suggested that NPSR1-AS1 upregulation may be a common event.

### 3.3. Functional Enrichment Analysis

**Figure 3:**
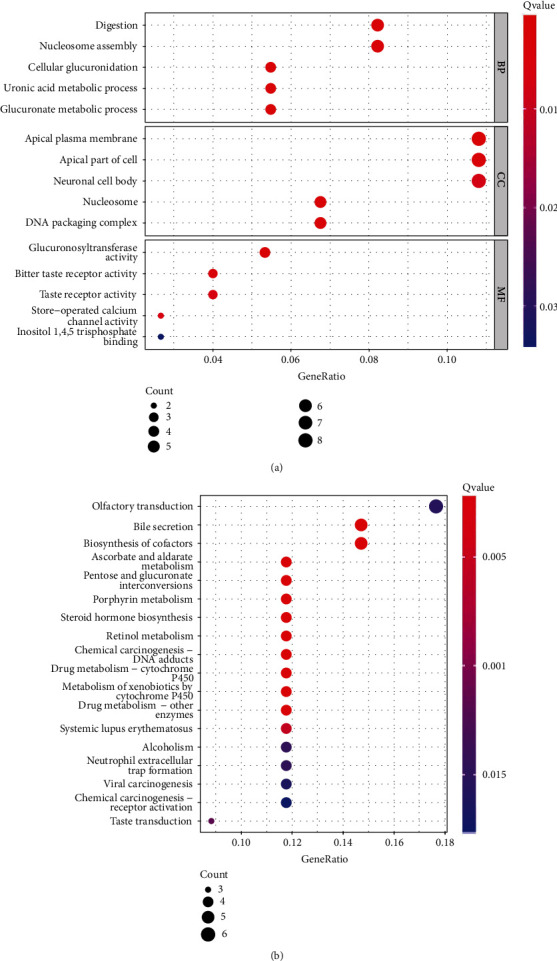
Functional enrichment analysis of DEGs between the high-NPSR1-AS1-expression group and low-NPSR1-AS1-expression group. (a) Significantly enriched GO terms of DEGs. (b) Significant KEGG pathway terms of DEGs.

To further explore the roles of NPSR1-AS1 in LUAD, we used the “limma” R package to separate the tumor groups into high- and low-expression subgroups based on median expression values of NPSR1-AS1. Following that, 79 DEGs were found. Then, we performed GO analysis using 79 DEGs. As shown in Figure 3(a), we found that the 79 DEGs were mainly enriched in digestion, nucleosome assembly, cellular glucuronidation, uronic acid metabolic process, glucuronate metabolic process, apical plasma membrane, apical part of cell, neuronal cell body, nucleosome, DNA packaging complex, glucuronosyltransferase activity, bitter taste receptor activity, taste receptor activity, store-operated calcium channel activity, and inositol 1,4,5-trisphosphate binding. Moreover, the results of KEGG assays indicated that the 79 DEGs were mainly enriched in olfactory transduction, bile secretion, biosynthesis of cofactors, ascorbate and aldarate metabolism, pentose and glucuronate interconversions, porphyrin metabolism, steroid hormone biosynthesis, retinol metabolism, viral carcinogenesis, and chemical carcinogenesis-receptor activation (Figure 3(b)).

### 3.4. Correlation of High NPSR1-AS1 Expression with Clinicopathological Features of LUAD

**Figure 4:**
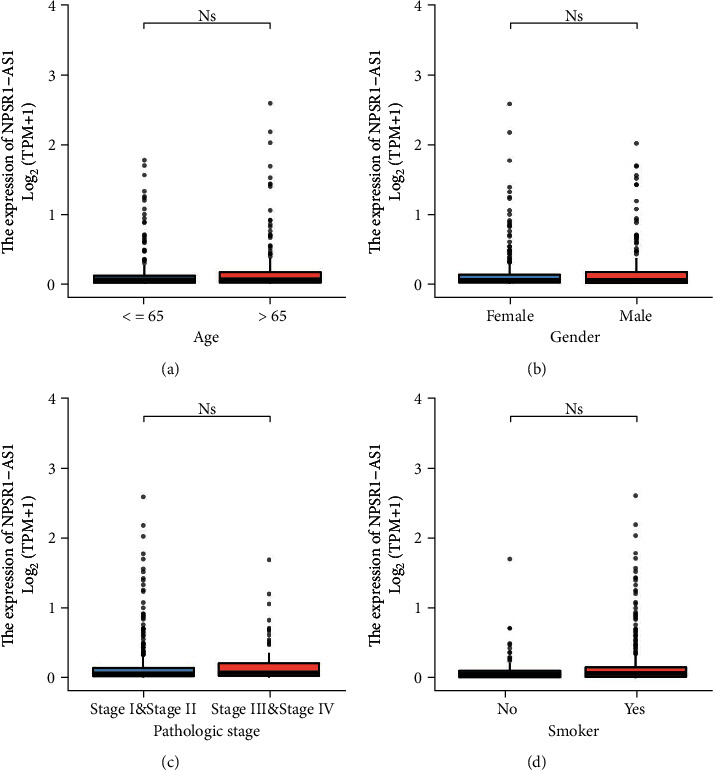
Relationships between NPSR1-AS1 expressions and clinicopathological parameters in LUAD patients. (a) Age. (b) Gender. (c) Pathologic stage. (d) Smoker.

In order to carry out statistical analysis, the level of NPSR1-AS1 expression was split between high- and low-expression groups. In individuals diagnosed with LUAD, an investigation was conducted to determine whether or not there was a correlation between the expression of NPSR1-AS1 and clinicopathological features. However, we find that the expressions of NPSR1-AS1 were not associated with age (Figure 4(a)), gender (Figure 4(b)), pathologic stage (Figure 4(c)), and smoker (Figure 4(d)). In addition, the results from the chi-square test also showed a similar finding (Table 1).

### 3.5. Relationship between NPSR1-AS1 Expression and Survival Outcomes in LUAD Patients

**Figure 5:**
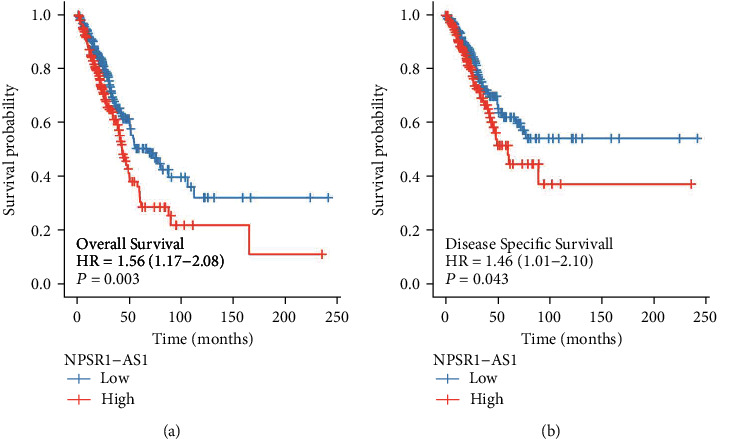
Kaplan-Meier survival analysis was applied to examine the prognostic value of NPSR1-AS1 expression in (a) OS and (b) DSS of LUAD patients.

Further, we investigated whether or not the expression of NPSR1-AS1 was connected with the fate of LUAD patients. Patients who had high levels of NPSR1-AS1 had a lower overall survival rate than those who had low levels of NPSR1-AS1 (Figure 5(a), *P* = 0.003), as shown by the findings of a Kaplan-Meier survival analysis. In addition, the group with high levels of NPSR1-AS1 showed a considerably lower DSS than the group with low levels of NPSR1-AS1 expression (Figure 5(b), *P* = 0.043).

### 3.6. Prognostic Factors Determined by Univariate and Multivariate Cox Regression Analysis

The next step was to conduct univariate and multivariate analysis to determine whether the NPSR1-AS1 expression level was an independent predictive indicator of LUAD patient outcomes. According to the findings of our study, both the pathologic stage and the expression of NPSR1-AS1 functioned as independent prognostic indicators for overall survival (Table 2). In addition, it was demonstrated that the pathologic stage is an independent prognostic indication for patients diagnosed with LUAD (Table 3). However, no additional evidence of NPSR1-AS1 expression could be found in DSS (Table 3).

### 3.7. The Expression of NPSR1-AS1 Was Associated with Immune Cell Infiltration

**Figure 6:**
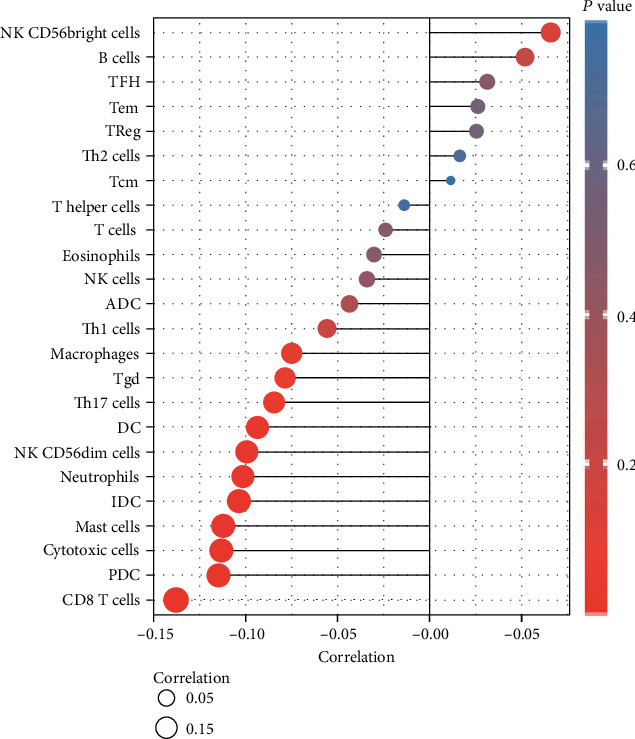
The expression of NPSR1-AS1 was associated with immune cell infiltration.

The ssGSEA methodology was utilized to analyze the transcriptomes of TCGA-LUAD cohort in order to determine the degree to which immune cell infiltration was present. Twenty-four immune-related phrases were included in the study in order to determine the number of immune cells that are present in the microenvironment of a tumor. Our group observed that the expressions of NPSR1-AS1 were negatively associated with CD8 T cells, pDC, cytotoxic cells, mast cells, iDC, neutrophils, NK CD56dim cells, DC, Th17 cells, Tgd, and macrophages, while they were positively associated with NK CD56bright cells and B cells (Figure 6).

## 4. Discussion

Tumor developments were dependent on the survivals and death of tumor cells [31]. The study of cell death can therefore assist us in understanding the underlying mechanisms that are responsible for the development of malignancies [32]. In addition to the well-known techniques of functional genes, researchers are uncovering other kinds of regulators that are involved in the progression of tumors. In recent years, for instance, lncRNAs have garnered a lot of attention from researchers [33, 34]. Research on lncRNAs has also become increasingly common. However, the majority of the attention has been directed toward conducting more in-depth basic studies. The question of whether lncRNAs can give doctors with some therapeutic insight has received very little attention in the published research. In the subject of LUAD research, there are likewise very few studies. Therefore, in the hopes of locating additional new approaches that may be utilized for clinical diagnosis and therapy, we decided to conduct research on the relationship that existed between lncRNAs and the clinical data associated with LUAD.

In our study, we performed an analysis on TCGA datasets, and as a result, we found a total of 229 DELs, which included 216 upregulated lncRNAs and 13 downregulated lncRNAs. NPSR1-AS1 was the primary focus of our attention. In the past, a number of studies have indicated that NPSR1-AS1 served a function in a variety of cancers. For instance, He et al. revealed that the expressions of NPSR1-AS1 were shown to be increased in hepatocellular carcinoma tissues and cell lines. In the following step, the ectopic expression of NPSR1-AS1 regulated the MAPK/ERK pathway, which in turn accelerated the proliferation and glycolysis of hepatocellular carcinoma cells [35]. Dastjerdi et al. showed that NPSR1-AS1 had the ability to make a considerable distinction between the tumor and the normal samples. These findings might have repercussions for the early diagnosis and focused treatment of colorectal cancer in the future [36]. He et al. discovered that NPSR1-AS1 activated the MAPK pathway to promote the proliferation and metastasis of thyroid cancer cells by engaging ELAVL1 to stabilize NPSR1 mRNA. This was accomplished by facilitating the proliferation of thyroid cancer cells [35]. In the first part of our study, we observed that the level of NPSR1-AS1 was significantly higher in LUAD tissues compared to nontumor specimens. This finding was in line with findings from other studies. The findings of the ROC tests then revealed that NPSR1-AS1 may be utilized as an indicator to screen LUAD specimens vs. nontumor specimens. In addition, the expression of NPSR1-AS1 was shown to be significantly elevated in many other kinds of tumors, such as CHOL, COAD, and ESCA, according to the findings of a pancancer investigation. Our research led us to believe that an upregulation of NPSR1-AS1 is a rather typical occurrence. The GO and KEGG tests found evidence that NPSR1-AS1 may play a regulatory role in the course of LUAD by exerting an influence over a number of different tumor-related pathways. Patients who had a high level of NPSR1-AS1 expression in clinical studies were found to have a lower overall survival time and disease-free survival time than patients who had a low level of NPSR1-AS1 expression. Moreover, multivariate studies demonstrated that NPSR1-AS1 expression was an independent prognostic factor for overall survival of LUAD patients. Based on these findings, we hypothesized that NPSR1-AS1 could serve as a diagnostic and prognostic biomarker for patients with LUAD. The prognosis of LUAD will be further investigated in subsequent studies in which we will also further evaluate the association between NPSR1-AS1-associated genes and the prognosis.

Tumor stromal cells are part of the tumor microenvironment and can influence how cancerous tumor cells behave [37]. One type of immune cell that plays a crucial role in tumor development and progression is called tumor-infiltrating lymphocytes (TILs). By building a complex intercellular contact network, TILs aid in the development and maintenance of an immunosuppressive environment, aid in immune escape, and eventually contribute to tumor progression [38]. Research on immune cell infiltration has revealed that there is a significant function for immune cells in the TME in the progression of cancer. New approaches to cancer immunotherapy may be easier to come up with if researchers had a better grasp of how immune cells infiltrate the immunological milieu. We found that the expression of NPSR1-AS1 was negatively associated with CD8 T cells, pDC, cytotoxic cells, mast cells, iDC, neutrophils, NK CD56dim cells, DC, Th17 cells, Tgd, and macrophages, while it was positively associated with NK CD56bright cells and B cells. It is possible that Th17 cells have an antitumor effect because the subgroup of patients with LUAD that has a greater infiltration of Th17 cells is less likely to develop lymph node metastases and more likely to have a better prognosis. Therefore, based on the findings of our study, immunosuppression, which is caused by the presence of less Th17 cells in the primary tumor microenvironment, may be the cause of a shorter survival rate at 10 years for patients with LUAD who have high levels of NPSR1-AS1.

Despite the fact that our research showed a relationship between NPSR1-AS1 and LUAD, there were still several limitations to our investigation that need to be addressed. Firstly, the number of patients who participated in this study was rather low, which meant that additional research including a substantial number of participants was necessary to validate our findings. Secondly, most of our findings were obtained from bioinformatics analysis and TCGA datasets, which lack experimental verification in in vitro and in vivo experiments.

## 5. Conclusion

It is possible that NPSR1-AS1 is a predictive biomarker for LUAD, which is the factor that determines how well cancer immunotherapy works. The findings of the current research have the potential to offer fresh perspectives on the formulation of efficient therapy methods directed against LUAD.

## Figures and Tables

**Table 1:** The relationship between NPSR1-AS1 expression and clinicopathological characteristics in patients with LUAD.

Characteristic	Low expression of NPSR1-AS1	High expression of NPSR1-AS1	*P*
*n*	267	268	
Age, *n* (%)			1.000
≤65	128 (24.8%)	127 (24.6%)	
>65	130 (25.2%)	131 (25.4%)	
Gender, *n* (%)			0.968
Female	142 (26.5%)	144 (26.9%)	
Male	125 (23.4%)	124 (23.2%)	
Pathologic stage, *n* (%)			0.244
Stage I	158 (30%)	136 (25.8%)	
Stage II	57 (10.8%)	66 (12.5%)	
Stage III	36 (6.8%)	48 (9.1%)	
Stage IV	12 (2.3%)	14 (2.7%)	
Age, median (IQR)	66 (59, 72)	66 (59, 72)	0.616

**Table 2:** Univariate and multivariate analysis of different prognostic factors for overall survival in patients with LUAD.

Characteristics	Total (*N*)	Univariate analysis		Multivariate analysis	
		Hazard ratio (95% CI)	*P* value	Hazard ratio (95% CI)	*P* value
Gender	526				
Female	280	Reference			
Male	246	1.070 (0.803-1.426)	0.642		
Age	516				
≤65	255	Reference			
>65	261	1.223 (0.916-1.635)	0.172		
Pathologic stage	518				
Stage I & stage II	411	Reference			
Stage III & stage IV	107	2.664 (1.960-3.621)	<0.001	2.535 (1.860-3.455)	<0.001
NPSR1-AS1	526				
Low	263	Reference			
High	263	1.557 (1.165-2.081)	0.003	1.442 (1.074-1.936)	0.015

**Table 3:** Univariate and multivariate analysis of different prognostic factors for disease specific survival in patients with LUAD.

Characteristics	Total (*N*)	Univariate analysis		Multivariate analysis	
		Hazard ratio (95% CI)	*P* value	Hazard ratio (95% CI)	*P* value
Gender	491				
Female	262	Reference			
Male	229	0.989 (0.687-1.424)	0.954		
Age	481				
≤65	243	Reference			
>65	238	1.013 (0.701-1.464)	0.944		
Pathologic stage	483				
Stage I & stage II	389	Reference			
Stage III & stage IV	94	2.436 (1.645-3.605)	<0.001	2.322 (1.562-3.450)	<0.001
NPSR1-AS1	491				
Low	251	Reference			
High	240	1.460 (1.013-2.105)	0.043	1.370 (0.943-1.988)	0.098

## Data Availability

The datasets used and analyzed during the present study are available from the corresponding authors upon rational request.

## Supplementary Materials

Supplementary Materials Table S1: the survival-related lncRNA in LUAD.
