# Supplementary material for: Comprehensive Analysis of NPSR1-AS1 as a Novel Diagnostic and Prognostic Biomarker Involved in Immune Infiltrates in Lung Adenocarcinoma
Source: J Oncol. 2022 Oct 15;2022:2099327. doi: 10.1155/2022/2099327 (PMC9588325; doi:10.1155/2022/2099327)
Supplement: Supplementary Materials — Table S1: the survival-related lncRNA in LUAD. [file 2099327.f1.docx]

**Table S1** The survival-related lncRNA in LUAD.

| Gene_name | p.Cox |
| --- | --- |
| PRKG1-AS1 | 6.58E-06 |
| AC087588.2 | 1.08E-05 |
| AC104971.3 | 4.43E-05 |
| MIR924HG | 5.39E-05 |
| AC093010.1 | 5.47E-05 |
| AL353746.1 | 5.93E-05 |
| HCG21 | 8.66E-05 |
| IER3-AS1 | 9.41E-05 |
| BANCR | 0.000105 |
| AF186192.1 | 0.000151 |
| MIR34AHG | 0.000162 |
| FAM83A-AS1 | 0.000163 |
| PPP1R14B-AS1 | 0.000173 |
| AC243960.1 | 0.000182 |
| VIM-AS1 | 0.000186 |
| SATB2-AS1 | 0.000193 |
| ITGB1-DT | 0.00021 |
| AC119424.1 | 0.000238 |
| AC026355.2 | 0.000251 |
| LINC01116 | 0.000259 |
| AC006369.1 | 0.000261 |
| SCAT1 | 0.000272 |
| COLCA1 | 0.000301 |
| CRNDE | 0.000308 |
| AC092171.5 | 0.000328 |
| OGFRP1 | 0.000343 |
| AC005479.2 | 0.000353 |
| AL353152.1 | 0.000364 |
| L3MBTL2-AS1 | 0.00037 |
| GAS6-AS1 | 0.00037 |
| AC079760.2 | 0.000382 |
| AL078645.1 | 0.00039 |
| AC022164.1 | 0.000411 |
| AC024257.1 | 0.000413 |
| AL445430.2 | 0.000427 |
| PRDM16-DT | 0.000437 |
| LINC01833 | 0.00045 |
| PP2672 | 0.00046 |
| LINC01132 | 0.000475 |
| AC131009.1 | 0.000494 |
| LINC02812 | 0.000499 |
| AC011444.3 | 0.000511 |
| LINC00861 | 0.000541 |
| LINC01031 | 0.000559 |
| AC106738.1 | 0.000574 |
| AC092718.4 | 0.000576 |
| AP000695.2 | 0.000576 |
| LINC02273 | 0.000585 |
| LINC00240 | 0.000587 |
| LINC00892 | 0.000602 |
| LASTR | 0.000615 |
| AL365259.1 | 0.000649 |
| AC021188.1 | 0.000651 |
| AC022784.1 | 0.000655 |
| AL606489.1 | 0.000658 |
| AC009226.1 | 0.000659 |
| LINC00639 | 0.000671 |
| ACVR2B-AS1 | 0.000678 |
| LINC02802 | 0.000682 |
| WWC2-AS2 | 0.000684 |
| SDK1-AS1 | 0.00071 |
| AC024884.2 | 0.000713 |
| TSPOAP1-AS1 | 0.000719 |
| LMO7DN | 0.00072 |
| LARGE-AS1 | 0.000725 |
| AC020765.2 | 0.00073 |
| LINC01117 | 0.000736 |
| AC007608.3 | 0.000742 |
| PDE6B-AS1 | 0.000776 |
| LINC02623 | 0.000789 |
| AL139041.1 | 0.000802 |
| AC048383.1 | 0.000809 |
| CEP250-AS1 | 0.000848 |
| MYRF-AS1 | 0.000854 |
| AC007384.1 | 0.000875 |
| AC092384.1 | 0.000878 |
| LINC02613 | 0.00088 |
| AC099850.3 | 0.000894 |
| AC078864.1 | 0.000901 |
| AC023796.1 | 0.000905 |
| GMDS-DT | 0.000935 |
| AL365203.2 | 0.000946 |
| LINC02611 | 0.000965 |
| LINC01819 | 0.000991 |
| PTCSC3 | 0.001001 |
| RBM38-AS1 | 0.001002 |
| GAS1RR | 0.001066 |
| LMO7DN-IT1 | 0.001067 |
| AC084855.1 | 0.001071 |
| SIAH2-AS1 | 0.001095 |
| AC021517.1 | 0.001101 |
| LINC00683 | 0.001109 |
| LINC02132 | 0.001112 |
| AC005759.1 | 0.001173 |
| AL691432.2 | 0.00125 |
| AL049874.3 | 0.001257 |
| AC092171.3 | 0.001327 |
| LIFR-AS1 | 0.001374 |
| AC007255.1 | 0.001414 |
| AL158151.2 | 0.00144 |
| LY86-AS1 | 0.00152 |
| AC012676.3 | 0.00154 |
| AC087752.3 | 0.001576 |
| AC091891.1 | 0.001581 |
| AL161781.2 | 0.001593 |
| SFTA3 | 0.001618 |
| LINC00494 | 0.00163 |
| AP003385.3 | 0.001643 |
| AC007378.1 | 0.001724 |
| AC011365.2 | 0.001725 |
| LINC00426 | 0.001731 |
| AC106738.2 | 0.001733 |
| AC068987.2 | 0.001741 |
| CADM3-AS1 | 0.001766 |
| AP000866.2 | 0.001783 |
| AL359220.1 | 0.001793 |
| LINC02723 | 0.001819 |
| AC023830.3 | 0.001842 |
| AC068228.1 | 0.001865 |
| MIR223HG | 0.001866 |
| AC108718.1 | 0.001871 |
| AC037441.1 | 0.001889 |
| LINC01451 | 0.001898 |
| AC005479.1 | 0.001913 |
| LINC01711 | 0.001932 |
| DRAIC | 0.001946 |
| AL133445.2 | 0.001971 |
| AC123912.4 | 0.002044 |
| FAM30A | 0.002059 |
| LINC01215 | 0.002128 |
| AC107959.1 | 0.002134 |
| MIR4435-2HG | 0.002147 |
| AL157895.1 | 0.00218 |
| WDR86-AS1 | 0.002217 |
| UGDH-AS1 | 0.002235 |
| CH17-340M24.3 | 0.002265 |
| AL391807.1 | 0.002267 |
| ELOA-AS1 | 0.002309 |
| AL662890.1 | 0.002331 |
| AL390755.1 | 0.00239 |
| AC011503.1 | 0.002405 |
| AC010343.3 | 0.002442 |
| AC018529.1 | 0.002497 |
| AC005908.2 | 0.002516 |
| ARHGEF26-AS1 | 0.002522 |
| C2orf91 | 0.002536 |
| AL360270.1 | 0.00256 |
| AC012368.1 | 0.002562 |
| AC004466.2 | 0.002589 |
| FAM215A | 0.002612 |
| AL049828.1 | 0.002619 |
| LINC00996 | 0.00266 |
| CASC8 | 0.002671 |
| AC097376.3 | 0.002733 |
| NPSR1-AS1 | 0.002771 |
| LINC02413 | 0.002773 |
| LINC02739 | 0.002797 |
| LINC02147 | 0.002818 |
| AC112206.2 | 0.002821 |
| AL118556.2 | 0.002835 |
| AC010186.4 | 0.002849 |
| LINC00857 | 0.00286 |
| AL033519.4 | 0.002874 |
| LINC02551 | 0.002886 |
| MIR99AHG | 0.002935 |
| LINC00907 | 0.002957 |
| Z83847.1 | 0.003052 |
| AC007952.6 | 0.00307 |
| AL139383.1 | 0.003119 |
| AC125603.3 | 0.003123 |
| LINC01913 | 0.003141 |
| AL133245.1 | 0.003159 |
| KDM2B-DT | 0.003174 |
| AL451069.1 | 0.003192 |
| AC068338.3 | 0.003198 |
| AC027288.3 | 0.00322 |
| AC091849.2 | 0.003233 |
| AL035587.1 | 0.003239 |
| AC007114.2 | 0.003242 |
| AL596087.2 | 0.003244 |
| AC011284.1 | 0.003246 |
| LINC01559 | 0.003247 |
| AC019069.1 | 0.003247 |
| AC002563.1 | 0.003262 |
| LSINCT5 | 0.00327 |
| HDAC2-AS2 | 0.003272 |
| AC021678.2 | 0.003284 |
| AC010329.1 | 0.003354 |
| LINC00578 | 0.003389 |
| GSEC | 0.003407 |
| LINC00663 | 0.003413 |
| AC079601.1 | 0.003429 |
| SLC25A5-AS1 | 0.003454 |
| AC236972.3 | 0.003467 |
| AL589745.1 | 0.003475 |
| AC068594.1 | 0.003481 |
| AC005486.1 | 0.003492 |
| LIX1-AS1 | 0.003492 |
| LINC02649 | 0.003497 |
| AP005131.2 | 0.003523 |
| ZSCAN16-AS1 | 0.003539 |
| AL512631.1 | 0.003571 |
| Z97353.2 | 0.003667 |
| AC123595.1 | 0.00368 |
| AC025917.1 | 0.003732 |
| ADAMTS9-AS1 | 0.003795 |
| SYNPR-AS1 | 0.003812 |
| LINC01740 | 0.003865 |
| AL136141.1 | 0.003975 |
| AL135999.3 | 0.003988 |
| LINC02568 | 0.004002 |
| LINC02397 | 0.00402 |
| AC005165.1 | 0.004108 |
| LINC00939 | 0.004163 |
| HCG14 | 0.004184 |
| AC009133.3 | 0.004227 |
| AC102953.2 | 0.004252 |
| AL162511.1 | 0.004261 |
| LINC02390 | 0.004275 |
| AC092809.2 | 0.004307 |
| AC004687.1 | 0.004322 |
| AC055855.2 | 0.004457 |
| AC138207.1 | 0.004519 |
| LINC02422 | 0.004549 |
| AL591767.1 | 0.004563 |
| AC022613.1 | 0.004566 |
| MIR155HG | 0.00458 |
| LINC00535 | 0.004586 |
| VPS9D1-AS1 | 0.004597 |
| LINC02709 | 0.0046 |
| AL021368.2 | 0.004603 |
| BZW1-AS1 | 0.00465 |
| LINC00652 | 0.004711 |
| AC006033.2 | 0.004733 |
| AC091132.2 | 0.004754 |
| FAM181A-AS1 | 0.004765 |
| AC005256.1 | 0.00479 |
| AC008957.1 | 0.004799 |
| GLIS2-AS1 | 0.004808 |
| LINC01480 | 0.004843 |
| AC012181.1 | 0.004864 |
| AC124248.1 | 0.004873 |
| AC007036.1 | 0.004885 |
| LINC00324 | 0.004892 |
| AC004066.1 | 0.004932 |
| LINC01843 | 0.004978 |
| REV3L-IT1 | 0.005008 |
| AC007728.2 | 0.005123 |
| AC125603.2 | 0.005151 |
| ZRANB2-AS2 | 0.005156 |
| AL365181.3 | 0.005177 |
| AL136366.1 | 0.005185 |
| AP003032.1 | 0.005193 |
| AC027228.2 | 0.005208 |
| TRG-AS1 | 0.005234 |
| AP000812.1 | 0.005243 |
| AC020659.1 | 0.005276 |
| LINC00528 | 0.005279 |
| SEC23A-AS1 | 0.005317 |
| C20orf197 | 0.005317 |
| MIR663AHG | 0.005319 |
| LINC00921 | 0.005335 |
| AP000763.3 | 0.005357 |
| AC080013.5 | 0.005385 |
| LINC02610 | 0.005399 |
| IFT74-AS1 | 0.005428 |
| AL365181.2 | 0.005443 |
| AC138356.1 | 0.005498 |
| AP005131.6 | 0.005527 |
| AC005586.2 | 0.005537 |
| AL163051.1 | 0.005541 |
| UMODL1-AS1 | 0.005635 |
| AL356481.1 | 0.005735 |
| AC068631.1 | 0.005811 |
| EMSLR | 0.005815 |
| AC125603.1 | 0.005817 |
| MMP2-AS1 | 0.005829 |
| AC020663.1 | 0.00583 |
| WWTR1-AS1 | 0.005836 |
| AC012181.2 | 0.005838 |
| AL121845.4 | 0.005852 |
| GCC2-AS1 | 0.005952 |
| AC040168.1 | 0.005973 |
| AL161785.1 | 0.005979 |
| AL357093.2 | 0.005983 |
| AC106820.4 | 0.006084 |
| AC092384.3 | 0.006125 |
| ADPGK-AS1 | 0.006183 |
| AP001972.3 | 0.006196 |
| AC026401.3 | 0.006213 |
| AC092071.1 | 0.006296 |
| LINC00402 | 0.006307 |
| AC015660.1 | 0.006385 |
| NAV2-AS2 | 0.006421 |
| AC011899.1 | 0.006433 |
| LINC02757 | 0.006439 |
| SH3BP5-AS1 | 0.006501 |
| AC104809.1 | 0.00653 |
| AC124242.1 | 0.006576 |
| AC009570.1 | 0.006636 |
| LINC01857 | 0.006637 |
| AC011477.1 | 0.006785 |
| SH3PXD2A-AS1 | 0.006796 |
| ZNF674-AS1 | 0.006799 |
| AC026471.2 | 0.006811 |
| AL445493.3 | 0.006812 |
| AL391001.1 | 0.006919 |
| AP005131.4 | 0.006929 |
| STIM2-AS1 | 0.006964 |
| LINC02656 | 0.007007 |
| AC093525.7 | 0.00701 |
| AC034111.1 | 0.00702 |
| LINC02323 | 0.007045 |
| AL031600.2 | 0.007057 |
| ITIH4-AS1 | 0.007076 |
| AL353150.1 | 0.00709 |
| ILF3-DT | 0.007102 |
| AC027601.2 | 0.007117 |
| WDR11-AS1 | 0.007148 |
| AC234775.3 | 0.007194 |
| LINC01644 | 0.007242 |
| AP001462.1 | 0.007315 |
| AC079848.1 | 0.007484 |
| LINC02754 | 0.007496 |
| AC005034.3 | 0.007583 |
| AC104024.1 | 0.007626 |
| C9orf139 | 0.007728 |
| LINC00539 | 0.007804 |
| ELN-AS1 | 0.007815 |
| AC107884.1 | 0.007821 |
| AC025278.1 | 0.007978 |
| AP000695.1 | 0.007997 |
| TMEM18-DT | 0.008046 |
| AC005037.1 | 0.008069 |
| AC022028.2 | 0.008086 |
| AL590666.2 | 0.008249 |
| HLA-DQB1-AS1 | 0.008269 |
| CCDC13-AS1 | 0.008306 |
| AC005884.1 | 0.008324 |
| AC087501.4 | 0.008358 |
| PRECSIT | 0.008376 |
| AC063977.6 | 0.00843 |
| TTC3-AS1 | 0.008437 |
| LINC02861 | 0.008492 |
| AC008738.2 | 0.008504 |
| RNASEH1-AS1 | 0.008506 |
| AL158835.1 | 0.008537 |
| LINC01624 | 0.008726 |
| AL139020.1 | 0.008796 |
| MAFG-DT | 0.008806 |
| LINC02576 | 0.008824 |
| MED4-AS1 | 0.008826 |
| AC018462.1 | 0.008843 |
| LRRC8C-DT | 0.008901 |
| AC008669.1 | 0.008906 |
| AC018926.3 | 0.008942 |
| AL031666.1 | 0.00897 |
| AL132656.3 | 0.008984 |
| AL033523.1 | 0.008986 |
| ZEB2-AS1 | 0.008989 |
| DELEC1 | 0.009 |
| AC012557.1 | 0.009016 |
| PRKAG2-AS1 | 0.009087 |
| SLCO4A1-AS1 | 0.009138 |
| AC004233.1 | 0.009159 |
| AC104984.3 | 0.009183 |
| AL139385.1 | 0.009193 |
| LINC01561 | 0.009203 |
| AC022167.3 | 0.009248 |
| LINC00973 | 0.009307 |
| LNCOC1 | 0.009328 |
| ZNF433-AS1 | 0.00943 |
| LINC02732 | 0.009479 |
| AC087071.1 | 0.009481 |
| AC073896.2 | 0.009562 |
| SLC2A1-AS1 | 0.009652 |
| AC012615.1 | 0.009695 |
| AL683813.2 | 0.009884 |
| MCCC1-AS1 | 0.009893 |
| CACNA1C-AS2 | 0.009922 |
| AC027288.1 | 0.009959 |
| LINC02535 | 0.009964 |
